# Estimating the effect of SNP genotype on quantitative traits from pooled DNA samples

**DOI:** 10.1186/1297-9686-44-12

**Published:** 2012-04-17

**Authors:** John M Henshall, Rachel J Hawken, Sonja Dominik, William Barendse

**Affiliations:** 1 CSIRO Livestock Industries, FD McMaster Laboratory Chiswick, Armidale 2350, NSW, Australia; 2 Cooperative Research Centre for Beef Genetic Technologies, University of New England, Armidale NSW 2351, Australia; 3 CSIRO Livestock Industries, Queensland Bioscience Precinct, Brisbane QLD 4067, Australia; 4 Present Address: Cobb-Vantress, Siloam Springs, Arkansas, USA

## Abstract

**Background:**

Studies to detect associations between DNA markers and traits of interest in humans and livestock benefit from increasing the number of individuals genotyped. Performing association studies on pooled DNA samples can provide greater power for a given cost. For quantitative traits, the effect of an SNP is measured in the units of the trait and here we propose and demonstrate a method to estimate SNP effects on quantitative traits from pooled DNA data.

**Methods:**

To obtain estimates of SNP effects from pooled DNA samples, we used logistic regression of estimated allele frequencies in pools on phenotype. The method was tested on a simulated dataset, and a beef cattle dataset using a model that included principal components from a genomic correlation matrix derived from the allele frequencies estimated from the pooled samples. The performance of the obtained estimates was evaluated by comparison with estimates obtained using regression of phenotype on genotype from individual samples of DNA.

**Results:**

For the simulated data, the estimates of SNP effects from pooled DNA are similar but asymptotically different to those from individual DNA data. Error in estimating allele frequencies had a large effect on the accuracy of estimated SNP effects. For the beef cattle dataset, the principal components of the genomic correlation matrix from pooled DNA were consistent with known breed groups, and could be used to account for population stratification. Correctly modeling the contemporary group structure was essential to achieve estimates similar to those from individual DNA data, and pooling DNA from individuals within groups was superior to pooling DNA across groups. For a fixed number of assays, pooled DNA samples produced results that were more correlated with results from individual genotyping data than were results from one random individual assayed from each pool.

**Conclusions:**

Use of logistic regression of allele frequency on phenotype makes it possible to estimate SNP effects on quantitative traits from pooled DNA samples. With pooled DNA samples, genotyping costs are reduced, and in cases where trait records are abundant this approach is promising to obtain SNP associations for marker-assisted selection.

## Background

Genotyping with dense single nucleotide polymorphism (SNP) assays is now common for studies on humans, laboratory animals, wild animals and livestock. In all cases, power increases with increasing numbers of genotyped individuals. Recent studies on quantitative traits in humans and livestock suggest that, for many traits, genetic variation is mainly due to a large number of regions in the genome, each having a small effect [1,2]. This has led to the conclusion that much larger numbers of individuals need to be genotyped than was assumed previously. With the progress in technology, throughput and number of SNP on the genotyping assays have increased. The price of genotyping each SNP has come down by orders of magnitude but the price per individual assay has remained relatively constant. Consequently, for studies for which many phenotype records are available at a low cost, it is interesting to reduce the number of assays required by genotyping pooled samples of DNA.

Research on the statistical analysis of data from pooled DNA experiments has included sources and effects of errors in allele frequency estimates, e.g. [3-5], experimental design, e.g. [3,6], and methods for inferring phase and haplotype frequencies [7,8]. To date, studies using DNA pooling reported in the literature concern mostly disease traits for which the phenotype is binary (diseased, not diseased) or quantitative traits that are effectively converted to binary traits by selective DNA pooling of samples from extreme performing individuals [9-11]. Marker effects on the quantitative trait can be estimated from the allele frequencies in the selected DNA pools [9].

In this paper, we propose and demonstrate a more general method to analyze pooled DNA samples when the phenotype is a quantitative trait with a continuous distribution. The effect of an SNP on a quantitative trait is measured in the units of the trait. For quantitative traits and with DNA assayed on individuals, it is usual to regress phenotype on genotype and to estimate the effect of each haploid copy of the SNP allele. As with selective genotyping [12], this method produces biased estimates of SNP effects when data originates from pooled DNA. The method proposed and demonstrated here, uses logistic regression of genotype on phenotype as proposed by Henshall and Goddard. The effect of the SNP genotype on the phenotype is calculated as a function of the effect estimated in the logistic regression. The objective of this study is to demonstrate the method using a simulated dataset and show that estimates are subject to little bias. The method is also applied to a livestock dataset with genetic and environmental stratification, with a model that includes the relationship between sample pools (as suggested by Sham et al. [6]), estimated from all SNP, as a covariate.

## Methods

### Statistical methods

Each SNP was analyzed independently to estimate SNP effects and significance levels. Conceptually, for a single SNP, a pooled sample of DNA consists of multiple haploid copies of each of the two SNP alleles, and associated with each haploid copy is a contribution to the overall pooled phenotypes. Our aim was to estimate the effect of the ratio of SNP alleles in the pooled DNA on the pool of phenotypes. Each individual contributes two haploid DNA alleles to the pool and, assuming an additive model for SNP effects, half of its phenotype to the pool of phenotypes for each haploid DNA copy. The variance of the haploid phenotypes is half the variance of the diploid phenotypes (since the diploid phenotypes are the sum of two random haploid phenotypes). If the effects associated with the diploid SNP genotypes are (-α, 0, +α), and if we assume a normally distributed error with diploid variance σ_e_^2^, then the distribution of the haploid phenotypes is a mixture of two normal probability density functions *f *^- ^and *f *^+^, with means μ - α/2 and μ - α/2, and variance σ_e_^2^/2, where μ is one half the diploid population mean. Let Y be a vector of haploid phenotypes, and let Z*_i _*be a random variable that takes the value 1 if haploid DNA copy *i *contains the SNP allele associated with effect + α/2, and the value 0 if haploid DNA copy *i *contains the SNP allele associated with effect -α/2, and let π_i _be the probability that Z*_i _*= 1. Then, using the same derivation as Henshall and Goddard [13]

$$\begin{array}{ccc} \pi & =\frac{f^{+}}{f^{+}+f^{-}} & \\ & =\frac{\exp\left(\frac{-1}{\sigma_{e}^{2}}{\left(Y-\mu -\frac{\alpha }{2}\right)}^{2}\right)}{\exp\left(\frac{-1}{\sigma_{e}^{2}}{\left(Y-\mu -\frac{\alpha }{2}\right)}^{2}\right)+\exp\left(\frac{-1}{\sigma_{e}^{2}}{\left(Y-\mu +\frac{\alpha }{2}\right)}^{2}\right)}\\ & =\frac{\exp\left(\frac{2\alpha }{\sigma_{e}^{2}}\left(Y-\mu \right)\right)}{\exp\left(\frac{2\alpha }{\sigma_{e}^{2}}\left(Y-\mu \right)\right)+1}. \end{array}$$

This can be written as

$$\begin{array}{ccc} \log\left(\frac{\pi }{1-\pi }\right) & =\frac{2\alpha }{\sigma_{e}^{2}}\left(Y-\mu \right) & \\ & =g+bY \end{array}$$

where *b *= 2α/σ_e_^2 ^and *g *= -2αμ/σ_e_^2^. Both *g *and *b *can be estimated using standard logistic regression software, and the estimate of *b *is an estimate of the effect of the phenotype on $\log\left(\frac{\pi }{1-\pi }\right)$. Our ultimate goal is an estimate of *α*, the effect of a SNP allele on the phenotype, and we obtain this by noting that σ^2 ^= σ_e_^2 ^+ 2*p*(1-*p*)*α*^2^, where σ^2 ^is the diploid phenotypic variance and *p *is the frequency of one of the SNP alleles. Solving for α in terms of *b *and *p *gives

(1) $$\alpha =\frac{-1+\sqrt{1+2p\left(1-p\right)b^{2}\sigma^{2}}}{2p\left(1-p\right)b}.$$

When *p *= 0.5, this formula is very similar to that of Henshall and Goddard [13]. Including allele frequency *p *was not necessary in their analyses of paternal half-sib data, since within large families, the allele frequency of the paternally derived allele is always close to 0.5. In practice, a more general model $\log\left(\frac{\pi_{i}}{1-\pi_{i}}\right)=bY_{i}+X_{i}\beta +e$ is used, where X is an incidence matrix containing covariates or stratification levels applying to the individual animals contributing to each pool, β is the vector of effects estimated for these covariates and factors, and e is a random error term. Standard software packages for logistic regression also provide an estimate of the significance of the effect of the phenotype on the SNP allele frequencies. We used this value as our estimate of the significance of the effect of the SNP allele on the phenotype. All analyses were conducted in R [14] using the glm() function, with a binomial distribution for the error and a logit link function. In logistic regression, the dependent variable is a formulation of counts of the two alternative outcomes at each level of the explanatory variable. In pooled data, these counts are unobserved so they are derived by multiplying the estimated allele frequency for the pool by the number of individuals in the pool. The explanatory variable in the logistic regression is one half the vector of mean phenotypes for each pool. We evaluated two methods for the inclusion of the effect of contemporary group in the model: i.e. (1) pre-adjusting each individual's phenotype for contemporary group and (2) simultaneously fitting contemporary group and phenotype by including contemporary group as a factor in the incidence matrix X. For comparison, we tested the logistic regression method on genotype data from individuals as well as on genotype data from pools, and also estimated effects and significance for *α *by regressing individual phenotypes on genotypes (i.e. without pooling), using the model Y=Gα+Xβ+ε, where **G **is a vector of genotypes for individuals and ε is an error term. For this regression we used the R lm() function.

### Accounting for population stratification

Just as with individual samples, a potential problem in estimating SNP effects from pooled DNA is the presence of hidden population stratification [15]. To account for this, we used all the SNP to estimate a genomic correlation matrix for the pooled samples using the first of the three methods reported by VanRaden [16], except that instead of integer valued genotypes for individuals, real valued allele frequency estimates for pools were used with appropriate scaling. Then, the principal components of the genomic correlation matrix were used to account for stratification [17,18] by fitting the most important eigenvectors from the principal components analysis as covariates in the model when estimating the effect of a SNP and its significance. Since our aim is to compare the analysis of pooled samples with the analysis of individual samples, rather than to examine the effect of population stratification, we took the simple approach of always fitting the first five eigenvectors regardless of their significance. The eigenvectors were fitted along with contemporary groups, and we tested both pre-adjusting the data for both contemporary group and eigenvectors, and fitting the eigenvectors and contemporary groups concurrently with the SNP. Eigenvectors were not nested within a contemporary group, since our aim was to adjust equally for population stratification regardless of the level of genetic diversity within a contemporary group.

### Dataset A simulated

We simulated data on a population of 3000 individuals. No relationships between individuals were assumed and phenotypes consisted of the effect of a single SNP and an error term with variance σ^2^, which incorporates components due to environmental variation and all genetic effects other than the SNP under consideration. We repeated the simulation 1000 times, each time sampling and analyzing a single SNP. For each replicate, we sampled a population-wide frequency for the first SNP allele (*p*) from a uniform (0.01, 0.99) distribution and a population-wide SNP effect α from a uniform (0.0, 0.5) distribution. Diploid SNP genotypes were then sampled for each individual, and genotypic effects of 0, α or 2α were assigned to individuals with zero, one or two copies of the first SNP allele, respectively. Phenotypes comprised the genotypic effect plus a random error term, with a new error term for each individual sampled from a normal distribution with mean = 0 and variance σ^2 ^= 1.0. Individuals were ranked on phenotype and assigned to pools based on rank. To explore the effect of the number of pools, we used 2^n ^pools for n = 1:m, with m chosen such that 2^m ^was less than 3000, i.e. the number of individuals in the population. We calculated the mean phenotype and SNP allele frequency for each pool, with either no error simulated in the frequency estimates, or with an error component added to each allele frequency estimate. The error component was sampled from a normal distribution with mean = 0 and standard deviation = 0.05. Since frequencies are only meaningful when between 0 and 1, if adding the error resulted in an allele frequency that was less than 0 or greater than 1, frequencies were set to 0 or 1, respectively.

### Dataset B - beef cattle hip height

Records were available on 1041 female cattle born over three years and originating from two genetically diverse breeds, pure bred Brahman (*Bos indicus*) and Tropical composite (predominantly *B. taurus*). Tropical composite cattle are widely used in northern Australia, with founder genetics from a variety of breeds, including British *B. taurus *(e.g. Shorthorn, Hereford), but sometimes also Sanga (African *B. taurus*) and Brahman (*B. indicus*). We analyzed hip height (adjusted for age), measured in cm at around three years of age. Hip height is known to have a high heritability, for example Vargas et al. [19] estimated a heritability of 0.65 in Brahman cattle. Contemporary groups were a concatenation of measurement date, farm of origin and cohort, i.e., the interaction of these effects. All contemporary groups contained individuals of only one breed, so there was no need to explicitly include breed when assembling the groups. This resulted in 56 different groups, with 76 animals in the largest contemporary group and one animal in the smallest contemporary group. Groups with less than 16 animals were discarded, leaving 940 animals in 29 contemporary groups. We ranked animals on hip height phenotype within a contemporary group, and then allocated them to pools on the basis of rank within contemporary group, with approximately the same number of individuals in each pool. The smallest number of pools considered was 134 pools of six, seven or eight animals, since this made it possible to have two pools of eight animals for the smallest contemporary groups. To explore the effect of the number of pools, we also considered 268 pools of three or four animals, and 536 pools of one or two animals. Two additional analyses were conducted for comparison. First, pre-adjusting phenotypes for contemporary group and then ranking across contemporary group before allocating individuals to pools that took no account of contemporary group (and therefore breed) and second, using the 29 contemporary groups described above, but instead of pooling, using the genotype and phenotype of one randomly selected individual from each pool.

Individual genotypes for the BovineSNP50 Array [20] were available on each individual [21,22]. Pooled genotypes were generated *in silico *as the frequencies of alleles in animals in the pool. No random error term was added to the pool genotypes. For analyses that estimated SNP and contemporary group effects simultaneously, we checked the within contemporary group allele frequency for each SNP and excluded contemporary groups with minor allele frequencies less than 0.01 for that SNP. Essentially, this removes contemporary groups in which one SNP allele is fixed. The within contemporary group minor allele frequency for pooled data was estimated as the mean of the allele frequencies of the pools, weighted by the number of individuals in each pool. For analyses on data pre-adjusted for contemporary groups, we either included all contemporary groups for all SNP or excluded contemporary groups with minor allele frequencies less than 0.01 for the SNP under analysis.

## Results

For pooled data, we evaluated the logistic regression (LR) of genotype on phenotype using the significance of estimated SNP effects (expressed as minus log(p-value), abbreviated here by MLP) and the magnitude of the estimated SNP effect α, estimated using equation (1). In each case, the comparison is done with the MLP or α obtained using least squares (LS) regression of phenotype on genotype with SNP data from all individuals. For clarity in the presentation of results, generally we do not explicitly state the method (LR or LS). Where the data are referred to as "pooled" it is implicit that the analysis method is LR, even when the number of pools is equal to the number of individuals (since LR was also used on individual data). Where the data are referred to as "individual" it is implicit that the analysis method is LS. Selected comparisons are presented graphically, by plotting the MLP or α from pooled data against the MLP or α from individual data, for the 1000 replicates, in the case of simulated data, and for 1000 randomly selected SNP and all SNP with a significance value less than 1.0E-4, in the case of the cattle hip height data.

### Dataset A - simulated

**Figure 1:**
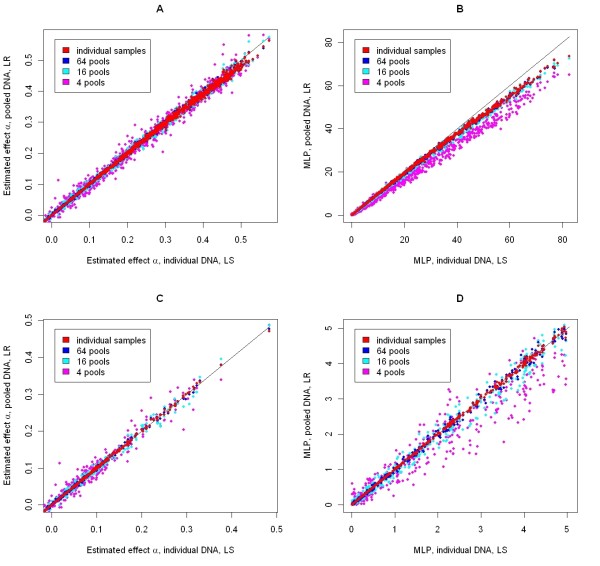
**Effects and significance for simulated SNP**. Estimated effects (A and C) and minus the log p-value (MLP, B and D) for 1000 replicates of a simulated SNP and simulated trait phenotypes; no genotyping error was simulated; effects or significance levels estimated using least squares regression of individual phenotype on individual genotype are on the X-axis, effects or significance levels estimated using logistic regression of pooled genotype on phenotype data are on the Y-axis; in (C and D), only SNP with p-values greater than 1E-5 are shown.

For the simulated data, summary statistics from various pooling scenarios are shown in Table 1. Both the coefficient of determination (R^2^) and the slope from a regression of MLP or α estimated from pooled data on the MLP or α estimated from individual data are presented. For the estimates obtained when no error in estimated allele frequency was simulated (Figure 1A), the estimate of α obtained from pooled data is plotted against the estimate of α from individual data. R^2 ^values obtained with 128 or more pools are practically identical to those with 3000 pools (i.e. individual samples, no pooling). Thus, only results for individual samples and for 64, 16 and four pools are shown graphically. With individual samples, the effect estimated using LR is smaller than the effect estimated using LS, indicating that the methods produce different estimates even when given the same (un-pooled) data. Most of the plotted points for 64 pools are masked by, or very close to those from individual samples (the order of plotting was from the smallest to the largest number of pools). As the number of pools decreases, the R^2 ^falls but the slope of the regression remains fairly constant. Figure 1B shows the MLP associated with the estimates of α in Figure 1A. For highly significant replicates, estimates from pooled data are less significant than those from individual data. Again, plotted points for 64 pools are masked by or very close to those for individual data. When the number of pools is reduced below 64, the significance of estimates from pooled data is reduced, as shown by the reduced slope of the regression in Table 1.

**Table 1:** Simulated data, regression of results from logistic regression of pooled data on results from least squares regression of individual data

Number of pools			3000	1024	512	256	128	64	32	16	8	4	2
**Parameter**	**Statistic**	**Subset**	**With no genotyping error simulated**										

α	R^2^	All SNP	0.998	0.998	0.998	0.998	0.998	0.998	0.998	0.996	0.994	0.987	0.957
		MLP < 5	0.999	0.999	0.999	0.999	0.999	0.998	0.997	0.992	0.983	0.955	0.849
	
	Slope	All SNP	0.967	0.967	0.967	0.967	0.966	0.967	0.967	0.967	0.966	0.962	0.965
		MLP < 5	0.997	0.998	0.998	0.997	0.998	0.996	0.995	0.989	0.979	0.980	0.993
	
MLP	R^2^	All SNP	0.999	0.999	0.999	0.999	0.999	0.999	0.999	0.998	0.997	0.993	0.978
		MLP < 5	0.999	0.999	0.999	0.999	0.998	0.997	0.993	0.983	0.964	0.903	0.686
	
	Slope	All SNP	0.923	0.923	0.922	0.922	0.922	0.921	0.918	0.909	0.886	0.819	0.622
		MLP < 5	0.997	0.997	0.996	0.997	0.998	0.994	0.991	0.971	0.920	0.825	0.618

			With genotyping error (SD = 0.05) simulated										

α	R^2^	All SNP	-	0.975	0.929	0.895	0.861	0.808	0.705	0.597	0.388	0.110	0.024
		MLP < 5	-	0.911	0.693	0.527	0.385	0.318	0.181	0.100	0.059	0.004	0.015
	
	Slope	All SNP	-	0.929	0.900	0.903	0.915	0.894	0.908	0.911	0.947	0.829	0.873
		MLP < 5	-	0.773	0.618	0.587	0.543	0.572	0.639	0.503	0.721	-0.370	-1.586

MLP	R^2^	All SNP	-	0.996	0.988	0.982	0.971	0.942	0.881	0.819	0.677	0.455	0.195
		MLP < 5	-	0.909	0.765	0.680	0.577	0.334	0.139	0.076	0.053	0.039	0.016
	
	Slope	All SNP	-	0.910	0.907	0.916	0.928	0.909	0.917	0.902	0.906	0.758	0.531
		MLP < 5	-	0.924	0.882	0.871	0.831	0.823	0.757	0.670	0.953	1.424	1.112

Simulated data, 3000 individuals, regression of results from logistic regression (genotype on phenotype, 3000 individuals or pooled data) on results from least squares regression (phenotype on genotype, 3000 individuals); parameters are the estimated effect (α) and minus the log p-value (MLP); the coefficient of determination (R^2^) and slope of the regression line are shown; results are presented for all SNP, or for SNP with MLP from individual data less than 5 (p-value > 1E-05), and with no genotyping error simulated, or with genotyping error (SD = 0.05) simulated

The high R^2 ^between results from pooled samples and individual samples is partly due to the wide range of SNP effects simulated in the data (from 0.0 to 0.5 phenotypic standard deviations). Figures 1C and 1D present the same data as Figures 1A and 1B, but focus on replicates with MLP less than 5.0 (p-value > 1E-05), for which there is most uncertainty about whether SNP effects are real or simply due to chance. For these replicates, estimates of α and significance levels for 64 pools are almost identical to those from individual data (R^2 ^for α and MLP 0.998 and 0.999 respectively, regression slope equal to 0.998 for both α and MLP). This means that the estimated effect and significance level for an SNP will be almost exactly the same regardless of whether all 3000 individuals are genotyped or 64 pooled samples of DNA are genotyped. Even with four pools only, SNP that are significant with individual samples will be identified as significant (R^2 ^= 0.9), and with 16 pools the precision will be sufficient for most purposes (R^2 ^= 0.98).

These are best-case results, since no error in estimating allele frequency for the pools was simulated. As expected, introducing an error with a standard deviation of 0.05 (a 5% error) reduces the correspondence between the LR and LS results (Table 1). This is particularly the case for fewer pools and when effect sizes are small (MLP < 5).

### Dataset B - beef cattle hip height

Minor allele frequency (MAF) affected results more for pooled samples than for individual data, probably because pooling reduces the number of genotype observations in each contemporary group. Consequently, results are presented for SNP with a population-wide MAF > 0.20, MAF > 0.05 and MAF > 0.01. To summarize results, we present the R^2 ^from the regression of the results from pooled data on the results from individual data. The results from individual data were obtained using concurrent estimation of contemporary group, population stratification and SNP effects, with data from contemporary groups excluded when the within-group MAF was less than 0.01. This model is more complicated to set up because which individuals are included in the analysis varies between SNP, but we judged that this model would be less affected by any confounding of contemporary group and SNP allele frequency.

Results for selected analyses are presented in Table 2. They show that the R^2 ^are lower than for the simulated data, and fall rapidly as the number of pools is reduced. In most cases, there is little difference between the R^2 ^for both breeds analyzed together and for each breed analyzed separately. The R^2 ^obtained from pre-adjusted data are generally similar to those obtained from concurrent estimation, except when SNP with a MAF < 0.05 are included. For both concurrent estimation and pre-adjusted data, estimates of α from pooled data in Brahman with a MAF > 0.01 (i.e., including SNP with a MAF < 0.05) have a very low correlation with estimates from individual data. Further analysis of the results revealed that this is due to a small number of SNP with a low MAF and with very large effects when estimated using LS on individual data, so the slope of the regression of α estimated from pooled data on α estimated from individual data is close to zero.

**Table 2:** Hip height data, R^2 ^between parameters estimated using logistic regression and least squares regression

			940 individuals		536 pools		268 pools		134 pools	
	**MAF**	**Breed**	**R^2^(α)**	**R^2^(MLP)**	**R^2^(α)**	**R^2^(MLP)**	**R^2^(α)**	**R^2^(MLP)**	**R^2^(α)**	**R^2^(MLP)**

Concurrent estimation, contemporary groups with MAF < 0.01 excluded from analysis	> 0.20	both breeds	0.99	0.99	0.94	0.90	0.84	0.75	0.68	0.53
		
		Tropical composite	0.98	0.98	0.93	0.89	0.82	0.72	0.64	0.50
		
		Brahman	0.99	1.00	0.94	0.92	0.86	0.78	0.67	0.53
	
	> 0.05	both breeds	0.96	0.97	0.92	0.89	0.84	0.74	0.69	0.52
		
		Tropical composite	0.95	0.96	0.91	0.87	0.81	0.71	0.64	0.48
		
		Brahman	0.97	0.98	0.92	0.90	0.83	0.76	0.61	0.51
	
	> 0.01	both breeds	0.95	0.97	0.92	0.88	0.84	0.73	0.68	0.51
		
		Tropical composite	0.81	0.95	0.70	0.87	0.59	0.70	0.38	0.47
		
		Brahman	0.02	0.90	0.02	0.80	0.01	0.64	0.01	0.38

Pre-adjusted, all contemporary groups included in analysis	> 0.20	both breeds	0.96	0.99	0.91	0.90	0.80	0.74	0.65	0.53
		
		Tropical composite	0.99	1.00	0.93	0.89	0.81	0.71	0.64	0.50
		
		Brahman	0.99	1.00	0.94	0.91	0.86	0.79	0.67	0.54
	
	> 0.05	both breeds	0.97	0.99	0.92	0.90	0.83	0.73	0.69	0.53
		
		Tropical composite	0.98	0.99	0.93	0.89	0.82	0.71	0.66	0.50
		
		Brahman	0.97	0.99	0.93	0.91	0.86	0.79	0.69	0.54
	
	> 0.01	both breeds	0.97	0.99	0.91	0.89	0.83	0.73	0.71	0.53
		
		Tropical composite	0.84	0.98	0.80	0.88	0.71	0.70	0.59	0.49
		
		Brahman	0.00	0.76	0.00	0.68	0.00	0.59	0.00	0.41

Pre-adjusted, contemporary groups with MAF < 0.01 excluded from analysis	> 0.01	both breeds	0.97	0.99	0.93	0.90	0.85	0.73	0.72	0.53
		
		Tropical composite	0.95	0.99	0.92	0.89	0.82	0.71	0.65	0.50
		
		Brahman	0.93	0.90	0.78	0.62	0.80	0.62	0.45	0.18

Concurrent estimation, one random individual from each pool	> 0.20	both breeds	1.00	1.00	0.38	0.23	0.10	0.04	0.02	0.01
		
		Tropical composite	1.00	1.00	0.36	0.21	0.09	0.04	0.03	0.01
		
		Brahman	1.00	1.00	0.36	0.23	0.10	0.05	0.00	0.00

Adjustment for contemporary group before pooling	> 0.20	both breeds	0.95	0.91	0.74	0.62	0.74	0.61	0.30	0.17

Beef cattle hip height data, 940 individuals, BovineSNP50 Array; coefficients of determination (R^2^) between parameters (effect (α) and minus the log p-value (MLP)) estimated using a logistic regression (genotype on phenotype, individual or pooled data) and least squares regression (phenotype on genotype, individual data); SNP effects were either estimated concurrently with contemporary group and population stratification effects, or estimated on data pre-adjusted for those effects; in all rows except for the last, pooling was within contemporary group; results are presented for SNP with minor allele frequency (MAF) greater than 0.20, 0.05 and 0.01, for combined breed analyses and within breed analyses

**Figure 2:**
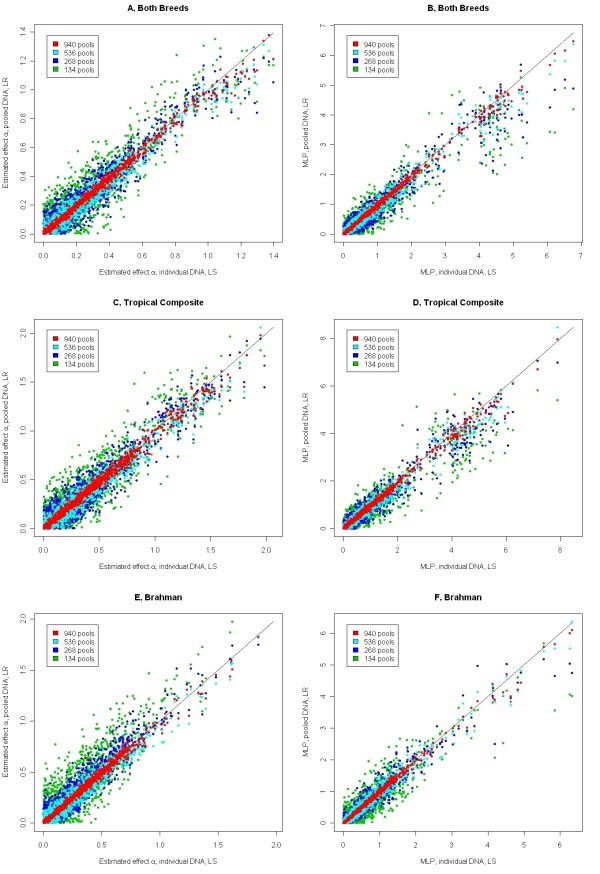
**Effects and significance for cattle hip height data**. Beef cattle hip height, estimated effects (A, C and E, in cm) and minus the log p-value (MLP, B, D and F) for 1000 randomly chosen SNP and for those with p-values less than 1E-4; effects or significance levels estimated using least squares regression of individual phenotype on genotype are on the X-axis, effects or significance levels estimated using logistic regression of pooled genotype on phenotype data are on the Y-axis; effects were estimated across breeds (A and B), within the Tropical composite breed (C and D) and within the Brahman breed (E and F).

To further illustrate the relationships that appear in the top three rows of Table 2 (MAF > 0.20 and concurrent fitting of contemporary groups, population stratification and SNP effects), estimated effects and significance levels are plotted in Figure 2. The correspondence between the methods is not as good as for the simulated data, especially when the number of pools is below 536. The ranking of estimated SNP effects or significance levels differs between pooled data and individual data. In particular, the most significant SNP are more significant with individual data or more pools, than with fewer pools, suggesting that pooling causes a loss of sensitivity.

Results obtained by analyzing a dataset with the phenotype and genotype of one randomly chosen individual from each pool are also presented in Table 2. When less than 100% of individuals are analyzed, for the same number of DNA assays, results from pooled data are far more correlated with results from individual data than are results from a subset of individuals.

We evaluated an alternative approach to the analyses described above, by pre-adjusting the phenotypes for contemporary groups, and then allocating animals to pools across breeds and contemporary groups. Clearly, this is not appropriate for such data because of the presence of two genetically divergent breeds; however a comparison of models is interesting since in some data, the existence of a genetic effect such as breed may not be known to the researcher. Even without pooling (i.e., 940 individual phenotypes and genotypes) and only including SNP with a MAF > 0.20, estimates were worse under this model than when pre-adjustment was performed after pooling (Table 2 R^2 ^of 0.91 and 0.99, respectively for MLP). For 940 individuals, these models differ only in the order in which fixed effects are fitted: the eigenvectors from the principal component analysis are identical. With less than 940 pools the eigenvectors from the principal components analysis differ according to whether pooling is performed before or after adjustment, and results for adjustment for contemporary groups before pooling are worse than for pooling within contemporary groups.

**Figure 3:**
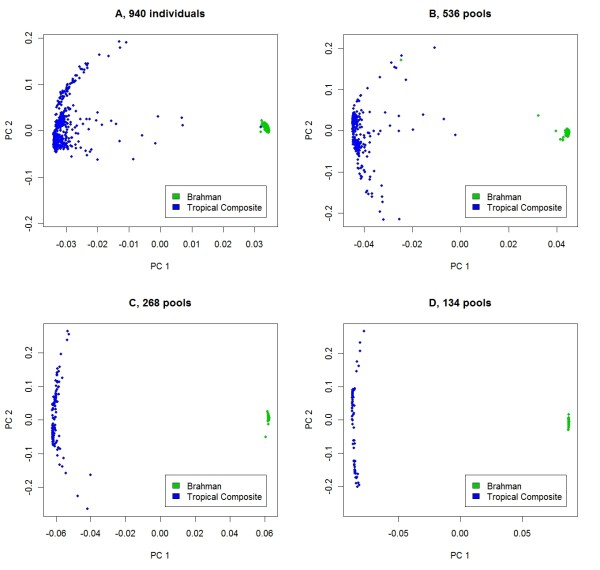
**Principal components of genomic relationships across two cattle breeds**. Plots of eigenvalues for the second principal component over eigenvalues for the first principal component from a genomic correlation matrix estimated using individual genotypes (**A**) or genotype data from 536 (**B**), 268 (**C**) or 134 (**D**) pools.

**Figure 4:**
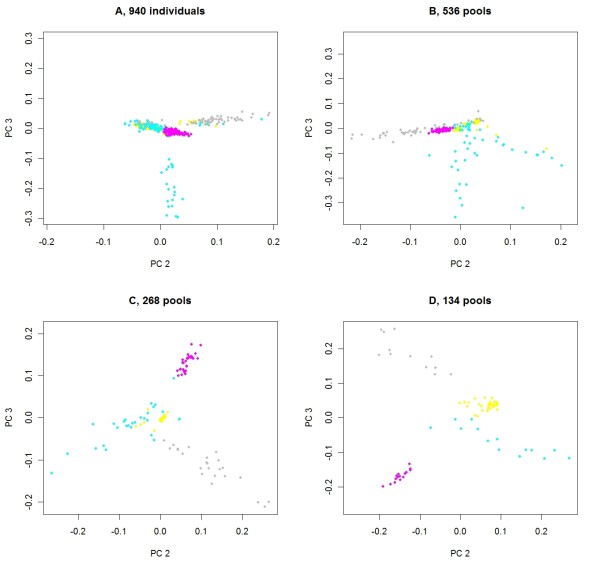
**Second and third principal components of genomic relationships for Tropical composite cattle**. For cattle from the Tropical composite breed, plots of eigenvalues for the third principal component over eigenvalues for the second principal component from a genomic correlation matrix estimated using individual genotypes (**A**) or genotype data from 536 (**B**), 268 (**C**) or 134 (**D**) pools; pools in (D) (134 pools) were colored on the basis of an ad hoc clustering, and when these pools were split to create 268 and 536 pools, or 940 individuals, pools retain the color of their "parent" pool.

**Figure 5:**
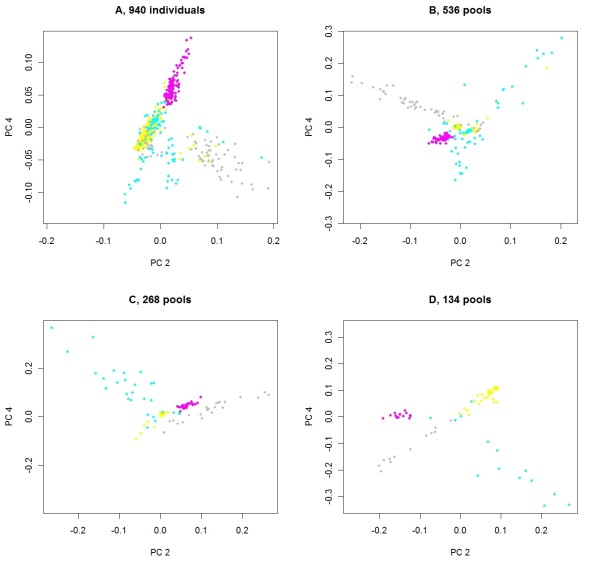
**Second and fourth principal components of genomic relationships for Tropical composite cattle**. For cattle from the Tropical composite breed, plots of eigenvalues for the fourth principal component over eigenvalues for the second principal component from a genomic relationship matrix estimated using individual genotypes (**A**) or genotype data from 536 (**B**), 268 (**C**) or 134 (**D**) pools; pools have the same color as in Figure 4.

To explore the effectiveness of using principal components to adjust for population stratification in pooled DNA samples, the first and second eigenvectors are plotted in Figure 3. Regardless of the number of pools, there is a clear separation between the Tropical composite and the Brahman populations. In Figure 4, the second and third eigenvectors for Tropical composite cattle are plotted. Clusters of pools were defined in an ad hoc manner for 134 pools (panel D). Then, when pools were split into 268, 536 or 940 pools, the new pools retain the color of the "parent" pool. Pools in the most distinct cluster (in pink) for 134 pools remain together regardless of the number of pools, but all become somewhat overlaid, and for 940 pools (panel A), there is little power to discriminate between pink, grey, yellow and half of the blue pools. For different numbers of pools, the principal components may explain different aspects of the variation. In Figure 5, the second and fourth eigenvectors are plotted with pools having the colors derived for the second and third eigenvectors, as in Figure 4. For 940 pools (panel A), the pink pools form a cluster characterized by high values for the fourth principal component and it is this cluster that is well characterized by the second and third principal components for 134 pools.

## Discussion

This study demonstrates that if SNP allele frequencies can be accurately measured in pooled samples of DNA, then SNP effects can be estimated for quantitative traits. Furthermore, in the datasets tested, these estimates are similar to those estimated when all individuals are genotyped. As the number of pools assayed increases, the estimates become more similar to those from individual genotype samples. With individual samples, the logistic regression of genotype on phenotype does not give identical results to least squares regression of phenotype on genotype. Thus, as the number of pools increases, estimates from logistic regression converge to a different value to that from least squares regression of phenotype on genotype. This is most notable for SNP of very large effect, where estimates from logistic regression are more conservative than those from LS. When no error in estimating pool allele frequencies is included, as for the simulated datasets used in this study, logistic regression with 128 pools per contemporary group produce almost identical estimates to those obtained using logistic regression with individual samples. There is no advantage in genotyping more than 128 pools per population, unless all individuals are genotyped and a regression of phenotype on genotype is performed. However, when an error (SD = 0.05) in estimating pool allele frequencies is introduced, the results are not nearly as good, and it is clear that controlling error will be very important in assembling pools and genotyping the pooled samples.

With the beef cattle hip height data, as the total number of pools increases, and therefore also as the number of pools per contemporary group increases, the results converge toward those for 940 pools (i.e. LR on individual data), suggesting that the strategy of pooling within contemporary groups and fitting contemporary group in the model is effective. This is important, as it may also be the most efficient way to organize an experiment. For example, it makes it possible to increase the size of an existing study by including estimated allele frequencies from pooled samples from additional contemporary groups, rather than having to pool the new samples with samples that have already been genotyped in the existing study. Furthermore, pre-adjusting the data for contemporary group and pooling based on adjusted phenotypes did not perform well in our study. In most cases, it made little difference whether phenotypes were pre-adjusted for contemporary group and stratification effects (after pooling) or whether estimation was done simultaneously. The only exception was for SNP with a small MAF and even then it was not the pre-adjustment itself that caused the difference, but the inclusion of data from contemporary groups in which only one SNP allele was present. A weakness of our analysis of the beef cattle data is that SNP allele frequencies for pools are estimated from individually assayed DNA samples, instead of from assays of pooled DNA. As such, our results are upper bounds for the performance of methods with pools, achievable only if the allocation of DNA to pools and subsequent assay introduces no error. However, our approach allowed us to consider a greater range of pooling strategies.

The motivation for assaying pooled samples of DNA rather than individual samples is to reduce costs. However, pooling DNA places restrictions on the experimental design and on the parameters that can be estimated, as discussed by Craig et al. [23] and others. For binary traits, it is difficult to include continuous covariates in the model. This is less of a problem with quantitative traits, as the individual phenotypes can be pre-adjusted for covariates, possibly using estimates from a larger dataset. The existence of population stratification has also been noted as a problem for pooled DNA analyses but in this study we successfully included information on the relationships between pools of individuals in the model. This is appropriate for the hip height data, since it is likely that some phenotypic similarities between pools are due to relatively close relationships. Many SNP that are not necessarily close to causal mutations may be in strong linkage disequilibrium with causal mutations due to recent common ancestry. The eigenvectors estimated using principal component analysis of the genomic correlation matrix show clear relationships to those from DNA from individuals (Figures 3, 4 and 5). An alternative to fitting these eigenvectors in the model would be to fit the correlation matrix itself as a random effect in the logistic regression estimation model.

With pooled DNA, only the effects of haploid SNP alleles can be estimated, so the estimates cannot be used in models with dominance or epigenetic (imprinting) effects. If SNP are sufficiently dense and all possible haplotypes are known in advance, then haplotype frequencies for pools can be estimated, as suggested by Barratt et al. [3]. The method described here assumes two haploid SNP alleles, which makes it unsuitable for SNP haplotypes, where more than two alleles are usually present. However, if the number of SNP in the haplotype is greater than the number of haplotype alleles in the population, then haplotype allele frequencies will be more accurately estimated than single SNP allele frequencies. These haplotype allele frequencies are easily collapsed down into estimates of SNP allele frequencies, suitable for use with the methodology proposed here.

For livestock quantitative traits, individual SNP effects are often small and an estimate of the breeding value or sum of the SNP effects for an animal is required. Methods currently used to estimate breeding values using high-density SNP genotype data apply distributional constraints to the estimated SNP effects to avoid over-fitting [24]. Some modifications to these methods will be required for pooled DNA, for which the genetic merit of selection candidates with individual DNA assays will be predicted from phenotyped individuals with pooled DNA. While the modifications are conceptually straightforward, they may pose computational problems. Analyses using the Gibbs sampler such as BayesA or BayesB [24] are already computationally demanding for large datasets, and fitting a logistic regression model requires more computer time than fitting least squares regression. However, with pooled DNA there are fewer observations to fit and it remains to be seen whether this reduction in data size will compensate for the increase in computational complexity. Our use of the genomic correlation matrix to correct for population stratification relates to another approach that is used to estimate genomic breeding values in livestock: the use of the genomic correlation matrix in place of a pedigree derived relationship matrix [25-27]. Under this model, there is no need to estimate the effects of individual SNP and this is probably a more computationally tractable approach for pooled data also.

## Conclusions

With pooled DNA samples, an estimate of the effect of an SNP on a quantitative phenotype can be estimated using a logistic regression of the pooled allele frequencies on the pooled mean phenotypes. For a simulated dataset with 3000 individuals and no error in estimating allele frequencies for pooled samples, there was little benefit in having more than 128 pools, unless all individuals were genotyped. With the livestock data, consisting of 940 individuals from two breeds distributed over 29 contemporary groups, having more pools was always an advantage. However, for a fixed number of assays, pooled samples produced far better results than one random individual assayed from each pool. Models that include the relationship between pools derived from a genomic correlation matrix can be used to account for hidden population stratification. Pooling across contemporary groups based on adjusted phenotypes with relationships between pools included in the model was for our data inferior to pooling within contemporary groups. This suggests that choosing the model and understanding and correctly fitting contemporary group structure are important in studies with pooled DNA data.

## Competing interests

The authors declare that they have no competing interests.

## Authors' contributions

JH conceived the study, participated in the design and statistical analysis of the study, and drafted the manuscript. RH carried out the molecular genetic analyses of the study, and participated in its design. SD contributed to the statistical analyses and helped to draft the manuscript. WB contributed to the conception of the study and participated in its design. All authors read and approved the final manuscript.
